# Quantifying Structure–Property Relationships in Ferroelectric Polymers Toward High‐Performance Soft Robots

**DOI:** 10.1002/advs.75884

**Published:** 2026-05-29

**Authors:** Ba Qin, Guotong Ding, Wanli Xing, Xiaoyu Yang, Wenxuan Li, Zhemin Chen, Shaobo Tan, Xiaoyong Wei, Zhicheng Zhang

**Affiliations:** ^1^ National Innovation Platform (Center) for Industry‐Education Integration of Energy Storage Technology School of Chemistry Xi'an Jiaotong University Xi'an Shaanxi P. R. China; ^2^ Electronic Materials Research Laboratory Key Laboratory of the Ministry of Education and International Center for Dielectric Research Xi'an Jiaotong University Xi'an P. R. China

**Keywords:** electromechanical response, ferroelectric polymers, P(VDF‐TrFE), soft robots, structure dependence

## Abstract

Owing to their excellent electromechanical (EM) response, poly(vinylidene fluoride‐trifluoroethylene) (P(VDF‐TrFE))–based ferroelectric polymers (FEPs) are extensively utilized in soft actuators. Currently, the strain (*S*_33_) of FEPs is mostly identified as electrostriction and described by *S*_33_ = *Q*_33_ *P* ^2^. Wherein, *Q*_33_ represents the electrostriction coefficient, *P* is polarization, and *Q*_33_ is mainly derived from data fitting. However, this approach fails to establish a connection between the composition and structure of FEPs, hindering the design of FEPs with higher EM response performance. This study introduces an effective model that quantitatively correlates the structural parameter interplanar spacing (*d*) to the EM response, namely *Q*_33_ = 100(Δ*d*/*d*_0_+1) × *Q*_33(s)_, where *d*_0_ = 4.31 Å, *Q*_33(s)_ = −0.54 m^4^/C^2^ are from single P(VDF‐TrFE). Guided by this model, we tailored the electrical properties and *d* of FEPs by incorporating 1,5‐Dihydroxy‐2,2,3,3,4,4‐Hexafluoropentane (HFPD), which results in a substantial improvement in the *S*_33_ by up to 100%. The composite films show promising application in fabricating high‐performance soft robots, including a biomimetic crawler (with a ultra‐fast crawling speeds of 27 cm/s) and a biomimetic butterfly (with a thrust‐to‐weight ratio of 0.71). Overall, our findings offer new insights for designing FEPs with superior EM responses, potentially driving notable advancements in flexible actuators.

## Graphical abstract

Here, we establish a quantitative relationship between the electrostriction coefficient (*Q*_33_) of FRPs and their interplanar spacing (d): *Q*_33_ = 100(Δ*d*/*d*_0_+1) × *Q*_33(s)_. Using this model, we fabricated high‐performance soft robots, including a biomimetic crawler (with a speed of 27 cm/s) and a butterfly (with a thrust‐to‐weight ratio of 0.71).

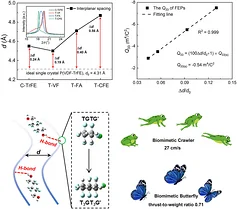

## 1 Introduction

Ferroelectric (FE) materials are widely used in capacitors [1, 2, 3], sensors [4, 5, 6], actuators [7, 8, 9, 10], and solid‐state refrigeration [11, 12, 13] owing to their excellent dielectric, ferroelectric, piezoelectric, and electrocaloric properties. In actuator applications, FE polymers (FEPs) such as poly(vinylidene fluoride‐trifluoroethylene) (P(VDF‐TrFE)) offer advantages over FE ceramics due to their flexibility and large strain (*S_33_*, −1% vs. 0.1%) [14, 15]. In addition, the higher output force makes FEPs more popular than dielectric elastomers in soft robotics, such as bionic insects [16]. Unlike dielectric elastomers based on Maxwell stress [17, 18], FEPs exhibit an excellent electromechanical (EM) response, which is closely related to their complex effect, comprising primarily the electrostriction effect (*S*_33_ = *Q*_33_ *P* ^2^) related to FE switching (FS) and conformation flipping (CF), and the inverse piezoelectric (IP) effect (*S*_33_ = *d*_33_ *E*) [19, 20, 21]. Here, *d*_33_ is the piezoelectric coefficient, *E* is the electric field, *Q*_33_ is the electrostriction coefficient, *P* is the polarization strength. Altering the chemical composition and FE phase structure of FEPs allows for tuning their EM properties over a wide range (−1% to −10%) [22]. However, accurately describing the EM response and clarifying the mechanisms in FEPs remain challenging owing to their inherent complex multi‐conformational components (mainly T >4, T_3_GT_3_G’, and TGTG’, where T and G/G’ represent trans and gauche conformations) [23, 24] and mixed‐phase structures (mainly composed of β, γ, and α) [25, 26].

For instance, the ideal P(VDF‐TrFE) single crystal comprises oriented T >4 conformations (β phase), forming a single aligned domain structure. Its EM response results mainly from the IP effect, which can be described by the single‐crystal model [27] (*d*_33_ = 2*Q*_33_ε_0_ε_r_ *P*_r_, *P*_r_ is remanent polarization). The actual P(VDF‐TrFE) primarily comprise long‐range T >4 conformations (∼95%) and exhibit a multidomain structure. Their EM response (*S*_33_) from FS and the IP effect is only around −1% due to the pseudohexagonal symmetry of P(VDF‐TrFE) unit cells [14]. To meet higher *S*_33_, chain conformation defects in P(VDF‐TrFE) introduced through electron irradiation [28] or the insertion of bulky third monomers [29, 30] can convert some T conformation into G/G’ ones and tailor the long T >4 conformation sequences. This strategy resulted in relaxor ferroelectrics (RFEs) mainly containing T_3_GT_3_G’ conformations (γ phase), with a large *S*_33_ exceeding −5% [15]. The high *S*_33_ in the RFEs primarily results from the CF (an electrostriction effect) from T_3_GT_3_G’ to T >4 driven by the external electric field, which can be estimated by *S*_33_ = *Q*_33_ *P* ^2^. However, the modified P(VDF‐TrFE) usually contain complex multi‐conformations depending on their compositions, and various physical effects may respond to E generating mechanical strain concurrently. Simply describing the EM response by electrostriction alone is unreasonable, as the nonlinear *S*_33_–*P* ^2^ relationship confirms [20]. In addition, the core electrical parameter *Q*_33_ has to be determined by linear fitting the experimental results and fails to explain the varying responses of FEPs with different components. Establishing the precise relationship between structure compositions and EM response in the FEPs remains challenging, markedly limiting deeper insights into EM response mechanisms in FEPs and the design of FEPs with optimal EM performance. In addition, the absence of direct relationship also restricts the design of FEPs with optimal EM performance for soft‐robots.

This study introduces a new model that incorporates multiple physical effects (FS, CF, and IP) and can handle EM response of FEPs with different compositions and complex conformations. This model takes the single‐crystal P(VDF‐TrFE) parameters [14, 31] (interplanar spacing *d*_0_: 4.31Å, electrostriction coefficient *Q*_33(s)_: −0.54 m^4^/C^2^, and IP coefficient *d*_33(s)_: −30 pm/V) as references. Four typical FEPs with different FE properties and EM response were investigated using the interplanar spacing change (Δ*d*/*d*_0_) caused by the different third monomer (Vinyl Fluoride (VF), Fluorinated Alkyne (FA), or Chlorofluoroethylene (CFE)) introducing into P(VDF‐TrFE) as the structural parameter. For the first time, we established the relationship between *Q*_33_ and structural parameter (Δ*d*/*d*_0_), where *Q*_33_ = 100(Δ*d*/*d*_0_+1) × *Q*_33(s)_ in P(VDF‐TrFE)‐based FEPs. Furthermore, guided by the model, we added HFPD into FEPs to tailor its electromechanical properties, and yielding a S_33_ of −2.0% at 50 MV/m. Thus, we fabricated high‐performance soft robots such as a biomimetic crawler that able to move at a ultra‐speed of 27 cm/s and a butterfly‐inspired robot with a thrust‐to‐weight ratio of 0.71. Our conclusion serves as a powerful tool for accurately understanding the complex EM responses of FEPs and provides precise guidance for designing high‐performance electroactive polymers.

## 2 Results and Discussion

### 2.1 Chain Structure and Composition

**FIGURE 1:**
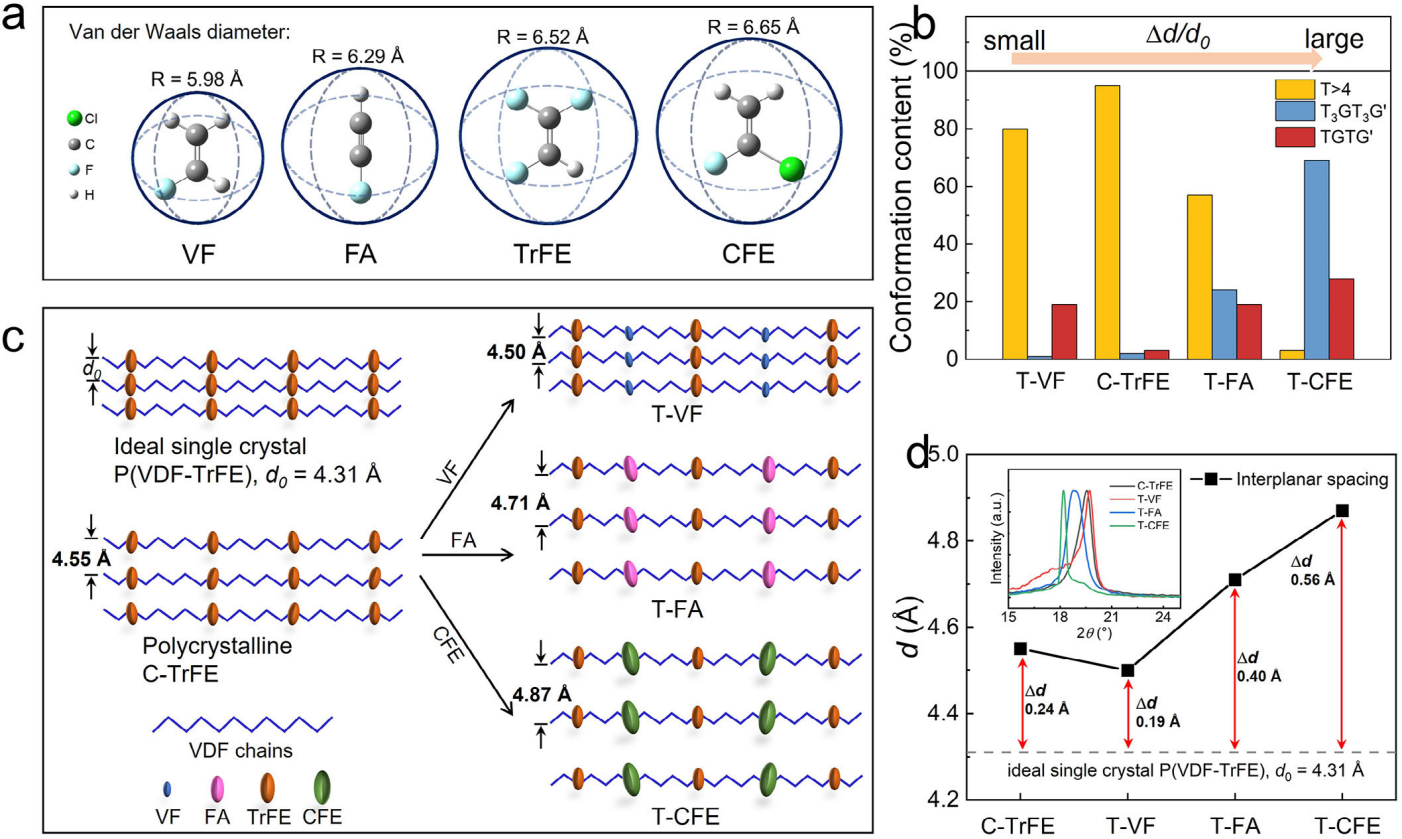
Chain structure and compositions. (a) the Van der Waals diameter of VF, FA, CFE units; (b) the conformations content of four polymers, the larger Δ*d/d*_0_, the higher content of T_3_GT_3_G’ conformation; (c) chain structure of single crystal P(VDF‐TrFE), C‐TrFE, T‐VF, T‐FA and T‐CFE; (d) the *d* of four polymers and their difference to ideal single crystal P(VDF‐TrFE), where Δ*d* = *d*_t_ − *d*_0_.

The study sample included four P(VDF‐TrFE)‐based FEPs with a fixed VDF/TrFE molar ratio (65/35) and ∼7 mol% third monomers with varied steric bulk. The complete elimination [32] and hydrogenation reaction [33] (Figures S1 and S2 and Table S1 and Methods) of the pristine terpolymer poly(vinylidene fluoride‐trifluoroethylene‐chlorofluoroethylene) (P(VDF‐TrFE‐CFE)) (with VDF/TrFE/CFE = 0.65/0.35/0.07, labeled as T‐CFE) afforded two terpolymers: P(VDF‐TrFE‐FA) with VDF/TrFE/FA = 0.65/0.35/0.07 (labeled as T‐FA) and P(VDF‐TrFE‐VF) with VDF/TrFE/VF = 0.65/0.35/0.07 (labeled as T‐VF). P(VDF‐TrFE) (VDF/TrFE = 0.65/0.35, abbreviated as C‐TrFE) was chosen for comparison. From the Van der Waals diameter [34] of VF, FA, TrFE, and CFE shown in Figure 1a, the steric volume of the monomers follows an increasing order of VF < FA < TrFE < CFE, which is designed to alter the chain conformation and the FE phase generated in the resultant terpolymers.

The third monomers can effectively regulate the chain conformation compositions of the P(VDF‐TrFE)‐based terpolymers. As shown in Figure 1b and Figure S3, C‐TrFE displays the predominant (∼95%) T >4 conformation (all‐*trans*, at 1288 cm^−1^ in FTIR), favoring normal ferroelectric phase (NFE) formation. Introducing VF leads to the formation of more TGTG’ conformation (614 cm^−1^) [35], while FA and CFE units result in more G conformation, including TGTG’ and T_3_GT_3_G’ (772 cm^−1^). Meanwhile, the third monomers more or less disrupt the typical all‐*trans* structure in C‐TrFE, which is correlated to their size. The smallest VF struggles to induce conformational disorder over a large spatial range in C‐TrFE, resulting in a slightly reduced T >4 conformation relative to P(VDF‐TrFE). Thus, T‐VF remains predominantly in the polar T conformation but forms more T >3 polar chain conformations (Figure S3). In contrast, the bulkier FA and CFE units can effectively promote the conformational disorder due to the struggle to enter the crystalline regions of the C‐TrFE chains. Therefore, T‐FA and T‐CFE form more G conformations, including TGTG’ and T_3_GT_3_G’, with a larger steric bulk reflecting a higher T_3_GT_3_G’ content. As the steric volume increases, the all‐*trans* conformation of the C‐TrFE gradually turns into TGTG’ and T_3_GT_3_G’ conformations, corresponding to the complexed NFE and eventually RFE, depending on the size and content of the third monomer.

The presence of third monomers alters the chain conformation content and *d* in the polymers. XRD data revealed a decrease in *d* in T‐VF but an increase in T‐FA and T‐CFE compared to C‐TrFE (Figure 1c). The decrease in *d* in T‐VF originates from the smaller steric volume of VF [36] and the increase in *d* in T‐CFE and T‐FA is ascribed to the large steric volume of CFE and the planar structure of the double bonds in FA [14]. For the complex composition and wide variety of FEPs, identifying the exact content of each conformation of the copolymers is always difficult. Since *d* is closely correlated to the chain conformation, composition, and the steric bulk of the monomers, Δ*d* = *d*_t_ *− d*_0_, referring to the difference between *d* of terpolymers (*d*_t_) and *d_0_* of P(VDF‐TrFE) single crystal (*d*_0_ = 4.31 Å) [14], is proposed to quantify the structural and compositional differences among different types of FEPs (Figure 1d and Figure S4). The reference P(VDF‐TrFE) single crystal displays the whole T >4 conformation or β phase with the smallest *d*, and the relative structural change (such as polycrystalline system or mixed phase) due to various compositions can be simply identified by Δ*d/d*_0_. In other words, the terpolymer with a smaller Δ*d/d*_0_ contains a more polar T conformation, whereas a larger Δ*d/d*_0_ refers to more G/G’ conformation compared to the reference P(VDF‐TrFE) single crystal (Figure 1b).

### 2.2 Conformational Evolutionary Behavior Under an Electric Field

**FIGURE 2:**
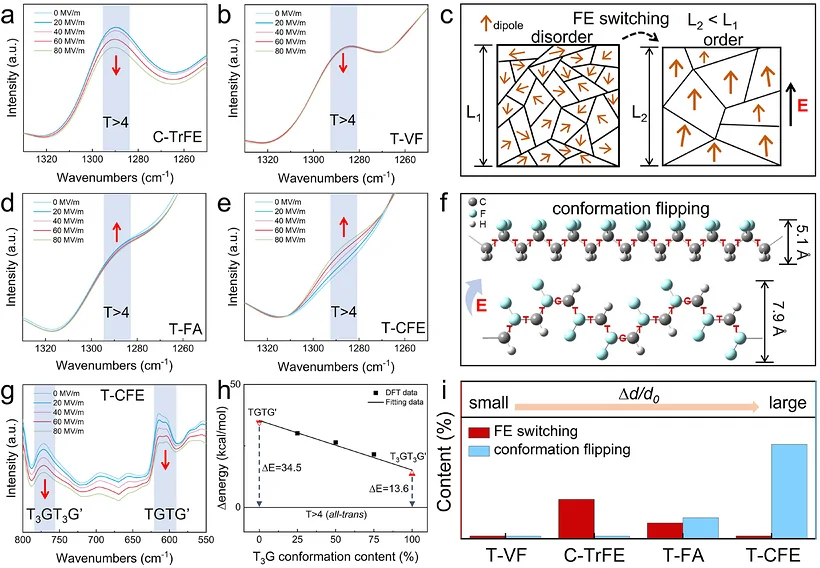
Conformation flipping under electric fields. (a) the conformation evolution behavior of T>4 in four polymers (a) C‐TrFE, (b) T‐VF, (d) T‐FA and (e) T‐CFE; the schematic diagram of FE switch (c) and conformation flipping (f); (g) the conformation evolution of T_3_GT_3_G’ and TGTG’ in T‐CFE; (h) the delta energy between TGTG’ and T_3_GT_3_G’ with *all‐trans* (setting *all‐trans* conformation as reference), where T_3_GT_3_G'content increases from 0 to 100%. (i) the relationship between EM response contribution and Δ*d/d*_0_.

The different conformations of the FEPs exhibited varied response behavior under the electric field, which could be identified by monitoring the dynamic evolution of the polymer chain conformations by in situ infrared spectroscopy under the gradient elevated electric field [37] from 0 to 80 MV/m (more information in methods). As the electric field increases, C‐TrFE and T‐VF show decreased T >4 conformation intensity while T‐FA and T‐CFE exhibit increased T >4 intensity (Figure 2a,b,d,e and Figures S5 and S6). Under an electric field, the T conformation is more stabilized than the G conformation, and the dipoles formed by the polar T >4 and T >3 conformations are more readily aligned along the direction of the electric field. Therefore, the reduction in the T >4 intensity in C‐TrFE and T‐VF results from FS‐induced anisotropy (Figure 2c) rather than an actual decrease in the T conformation content within the polymers. This FS causes the dipoles inside the material to align along the electric field direction, which only contributes to the weak EM response due to the pseudohexagonal symmetry of the β‐crystalline phase [14]. In addition, the almost unchanged T >4 conformation intensity in T‐VF than in C‐TrFE suggests that the polymer chain in T‐VF is more difficult to align under an electric field, which is closely related to its structure and composition (next part have details). In contrast, T‐FA and T‐CFE containing more G conformations can flip to form T conformations driven by the external electric field, manifesting as an increase in the T >4 conformation against electric fields [38]. Due to the notable differences in the chain width (Figure 2f), this CF is often considered the primary source of the high EM response in PVDF‐based FEPs [39].

Under elevated electric fields, the increase in the T >4 conformation intensity is always accompanied by a decrease in the intensity of the TGTG’ and T_3_GT_3_G’ conformations (Figure 2g, where T‐CFE is taken as an example). Usually, the flipping from TGTG’ to T_3_GT_3_G’ or from TGTG’ to T >4 requires a rather high external electric field (>300 MV/m or >500 MV/m) [22], which may not occur under an electric field below 100 MV/m. Therefore, the T_3_GT_3_G’ conformation should be the primary contributor to the transition to the T >4 conformation. DFT calculations also confirm this observation, as shown in Figure 2h (more information in methods), where higher T_3_GT_3_G’ content reflects a lower energy barrier in chains relative to the T >4 chains. The higher T_3_GT_3_G’ induced by adding bulky monomers with larger *Δd* readily favors CF from T_3_GT_3_G’ to T >4 under relatively low electric fields to produce a high EM response. However, a smaller Δ*d* leads to a higher TGTG’, which neither induces FS nor facilitates conformational transitions, may result in a reduced EM response. As discussed above, the CF of T_3_GT_3_G’ to T >4 and FS are the EM response sources in FEPs driven by the external electric field, which can be confirmed by two switching current (Figure S7). Beyond identifying the conformation and composition differences induced by various bulky monomers, Δ*d* could be employed to facilely correlate the chain structure differences and the contribution to the EM response in various FEPs. As shown in Figure 2i, T‐VF exhibits the smallest Δ*d*, making FS and CF difficult to occur. As Δ*d* increases, C‐TrFE undergoes primary FS with a weak EM response. As Δ*d* increases, the CF and FS in T‐FA are nearly even, indicating a markedly increased EM response compared to C‐TrFE. In contrast, the CF from T_3_GT_3_G’ to T >4 is almost entirely a response in T‐CFE, and the largest EM value is expected.

### 2.3 Dielectric and Ferroelectric Properties

**FIGURE 3:**
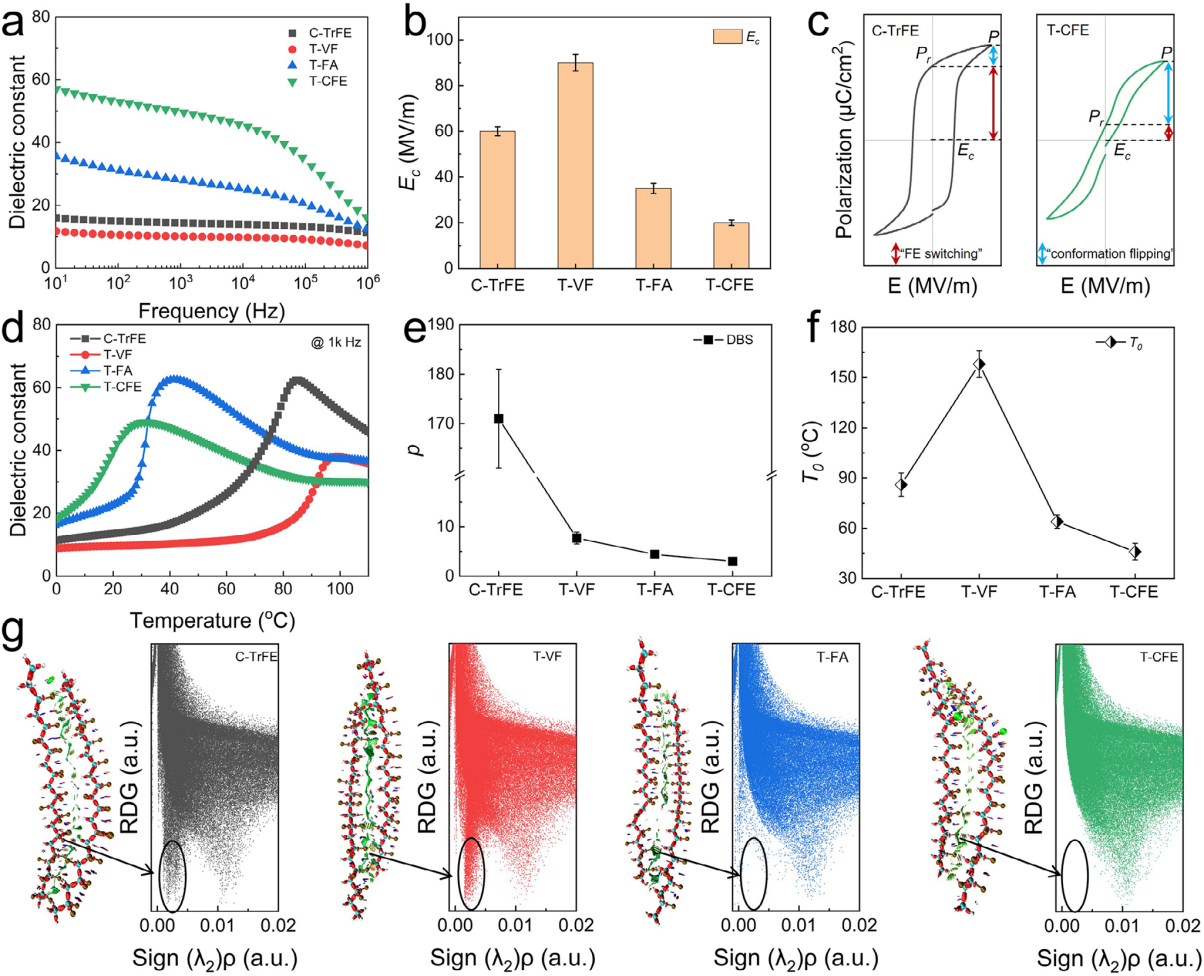
The electrical properties of four polymers. (a) the dielectric constant of four polymers at different frequency;(b) the *E*_c_ of four polymers; (c) the electric field dependence of “FE switching” and “conformation flipping”; (d) the temperature‐dependent dielectric constant of four polymers at 1 kHz; the *p* (e) and *T*_0_ (f) of four polymers; (g) the interchain interaction of four polymers calculated by DFT.

Incorporating different third monomers change the polymer chain conformation and alters the dielectric response under varying electric fields, which is also closely correlated to Δ*d*. As shown in Figure 3a, the four FEPs exhibit a similar decreasing trend against the testing frequency on the broadband dielectric spectra (BDS). The *ε*_r_ is in the order of T‐VF < C‐TrFE < T‐FA < T‐CFE, which is in the same order of Δ*d* value. Under elevated electric fields, C‐TrFE and T‐VF with smaller Δ*d* display the typical FE characteristic [40] with square *P–E* loops and large remanent polarization (*P*_r_) (Figure S8). T‐VF presents a lower *P*_r_ due to the half dipole moment of the VF unit compared with the VDF unit and the raised coercive electric field (*E*_c_) of 90 MV/m than the 50 MV/m of C‐TrFE (Figure 3b). However, the typical RFE [41] *P–E* loops with reduced *P*_r_ and *E*_c_ are observed in T‐FA and T‐CFE with larger Δ*d*, and their *E_c_* is much smaller than that of C‐TrFE and T‐VF. *P* under varying *E* is known to directly correlate with the chain conformation change along with the electric field‐driven FS. For C‐TrFE and T‐VF, when *E* < *E*_c_, the rapidly increased *P* is mostly from the FS of T >4 conformation (Figure 3c and Figure S9). When *E* > *E_c_*, FS approaches completion, the *P* increases slowly gradually due to dominant electronic polarization contributions. In contrast, the steadily increased *P* in T‐FA and T‐CFE mainly results from the CF from T_3_GT_3_G’ to T >4 (Figure 2). Apparently, adding a steric third monomer with larger Δ*d* gradually transitions the *P–E* loops from the normal ferroelectrics (NFPs) profiles of C‐TrFE to the RFE types of T‐FA and T‐CFE.

Temperature‐dependent BDS also confirmed the effect of steric bulky third monomers with different Δ*d* on the ferroelectricity of P(VDF‐TrFE)‐based copolymers (Figure 3d and Figure S10). C‐TrFE and T‐VF show typical FE profiles characterized by the independent peak *ε_r_* against frequency. In contrast, the maximus *ε_r_* of T‐FA and T‐CFE quickly shifted to the higher temperature as the frequency increased, exhibiting typical RFE behaviors [27]. Meanwhile, the peak dielectric constant temperature (*T*_max_, at 1 kHz) of the four polymers shows the same regularity with *E*_c_ property, agreeing well with the Δ*d* value. C‐TrFE exhibits a *T*_max_ of about 85°C; smaller VF units increase *T*_max_ to about 100°C, while bulky FA and CFE units reduce *T*_max_ to 40°C and 25°C, respectively. From the temperature‐dependent DBS spectra, the effect of different chain compositions on the relaxation strength of the system and the ease of FS within the material could be evaluated using the dielectric relaxation strength (DRS) formula: *lnf = lnf*_0_ *−(T*_0_ */T*_max_ *)^p^*, where *f* is the test frequency, *f*_0_ is the attempt frequency, *T*_0_ is the equivalent temperature of the activation energy, and *p* is DRS [42]. *p* tends to be infinity for NFPs, while it approaches 0.5 for RFEs. As shown in Figure 3e and Figure S11, the rather large *p* of C‐TrFE (∼170) suggests its NFPs characteristic. The continuously reduced *p* of T‐VF (∼30), T‐FA (∼5), and T‐CFE (∼2) indicates their increased RFE characteristic, which means adding third monomers would more or less decrease the NFPs characteristic of P(VDF‐TrFE), agreeing well with the chain conformation change discussed above.

In addition to chain conformation, adding the third monomer alters the interaction between polymer chains, thus affecting their electrical responses under various conditions. As shown in Figure 3f, T‐VF exhibits an abnormally high *T*_0_, along with high *Curie Temperature* (*T*_C_) and *E*_c_, although VF introduction disrupts the long‐range T >4 conformation structure of P(VDF‐TrFE) and reduces its ferroelectricity. Apparently, the notable increase in the activation energy required for FS indicates that factors other than conformation change affect the ferroelectricity of P(VDF‐TrFE)‐based polymers. To evaluate the effect of the third monomers on the interchain interactions, DFT theoretical calculations were conducted and visualized using the IRI + VMD program [43], as shown in Figure 3g (more information in methods). More points near 0.005 indicate stronger interactions [44]. Incorporating FA and CFE weakens interactions compared to C‐TrFE, while introducing VF strengthens them, making the FS require higher energy to occur, consistent with the observations in Figure 2b. Therefore, the ∆*d* resulting from the monomers’ steric volume primarily determines the polymers’ electrical response by shaping the interactions within and between polymer chains.

### 2.4 EM Response Performance

**FIGURE 4:**
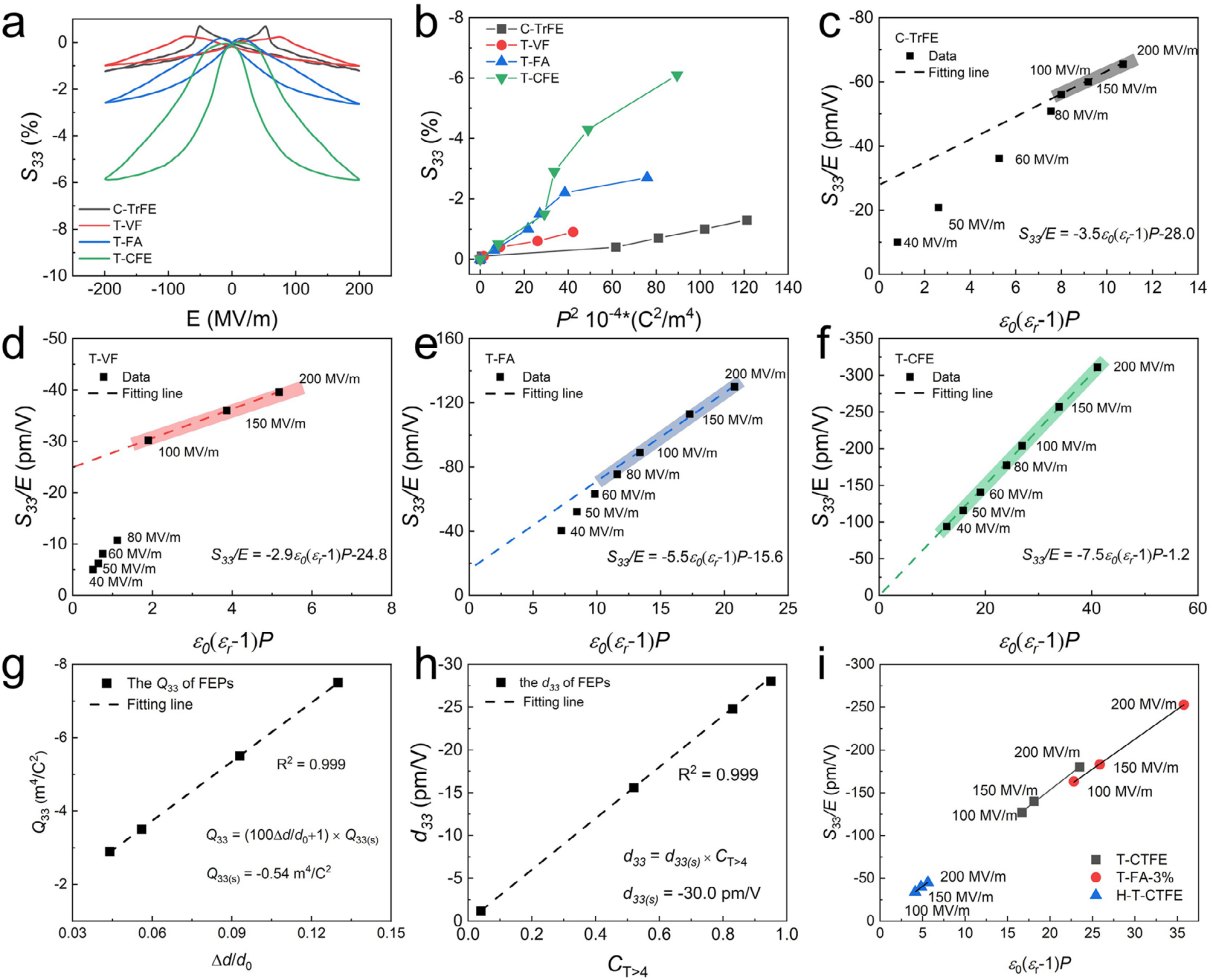
The EM response performance. (a) the *S*_33_ of four polymers at various fields; (b) the relationship of *S*_33_ and *P^2^* for four polymers, obviously it doesn't satisfy the linear relationship; (c) the fitting line of EM response(*S*_33_ */E*) and *ε_0_ε_r_P* in C‐TrFE (c), T‐VF (d), T‐FA (e) and T‐CFE (f) at various electric fields; (g) the fitting line of *Q*_33_ and Δ*d/d*_0_;(h) the fitting line of *d*_33_ and *C*_T>4_; (i) formulas are used to verify the EM properties of other FEPs.

Along with the change in permittivity and polarization under a varied electric field, adding bulky third monomers trigger a notable change in EM performance, which is correlated to the different polarization modes. Figure 4a and Figures S12 and S13 (Supporting Information) present the *S*_33_ curves of the four polymers as a function of *E*. Taking the results obtained at 200 MV/m as an example, C‐TrFE showed typical butterfly‐like curves with high hysteresis behavior, corresponding to FS stemming from its solely T >4 chain conformation. The hysteresis characteristic of T‐VF is slightly decreased due to the EM response of FS from the reduced T >4 conformation induced by VF addition. Both polymers show a rather low *S*_33_ of about −1.3% and −0.9%, suggesting the rather small contribution of FS to *S*_33_. In contrast, T‐FA and T‐CFE show the typical EM response of RFEs with slight or invisible electro‐strain hysteresis, and much higher *S*_33_ about −2.6% and −6.1% respectively, which could be ascribed to the huge contribution of their predominated CF from T_3_GT_3_G’ to T >4. This agrees well with the conformational change under the elevated electric field discussed in Figure 2. Therefore, adding bulky third monomers induced a chain conformation and interchain interaction change for the varied EM response, which was also closely correlated with the Δ*d* increase.

To quantitatively characterize the EM response of FEPs, the electrostriction model (*S*_33_ = *Q*_33_ *P* ^2^) is predominantly employed, which is oversimplified and does not consider the effect of structural composition [45]. Meanwhile, *Q*_33_ has to be obtained by linearly fitting the results of *S*_33_ and *P* ^2^, whose values could not quantitatively reflect the relationship to the chemical composition and the chain conformation of the polymers. As illustrated in Figure 4b and Figure S14, the relationship between *S*_33_ and *P* ^2^ for the four polymers under varying electric fields is presented using this model. C‐TrFE and T‐VF exhibit a good linear correlation between *S*_33_ and *P* ^2^, suggesting that the equation is suitable for NFPs. In contrast, T‐FA and T‐CFE, mostly in RFE, display poor linearity between *S*_33_ and *P* ^2^, which may be related to the complex chain conformations in RFEs corresponding to multi‐physics effects [46]. Crucially, *Q*_33_, as the key EM coefficient calculated from *S*_33_ = *Q*_33_ *P* ^2^, lacks explicit ties to the polymer structure and composition. This disconnection between the macroscopic parameters and the molecular architecture poses fundamental challenges for the precise modeling and predictive design of EM behavior in FEP systems.

To accurately describe the EM response of FEPs, contributions from three mechanisms have to be considered, including FS, CF and IP effect. First, in ferroelectrics, the strain from FS is a function of remnant polarizaiton (*P*_r_). Under the electric field below *E_c_*, *P_r_* is affected by not only the chemical composition of FEP but also many structural and fabrication factors including the thickness and annealing process. That makes it unlikely to establish a reliable relationship between *S*_33_ and *P*_r_ or Δ*d/d*_0_. However, above *E*_c_, the strain induced by FS could be facilely calculated since *P*_r_ is usually a constant depending solely on the chemical composition, which could be reliably correlated to Δ*d/d*_0_. Therefore, the strain contributed by FS above *E*_c_ can be approximated through the electrostriction effect [47]:

(1) $$S_{33}=Q_{33}\;Pr^{2}$$

Second, CF from T_3_GT_3_G’ to T>4(Figure 2e) is the major source of electrostriction effect in P(VDF‐TrFE)‐based FEPs, which can be calculated by the following Equation [20]:

(2) $$S_{33}=Q_{33}\left(P^{2}-Pr^{2}\right)$$

Thirdly, the origin and contribution of piezoelectricity in P(VDF‐TrFE)‐based ferroelectric polymers are quite complex^19^, but the IP is usually described as a linear function of *E* as follows:

(3) $$S_{33}=d_{33}E$$

Thus, by summing the three effects, the total EM response of P(VDF‐TrFE) based FEPs above *E_c_* can be described as follows:

(4) $$S_{33}=Q_{33}P^{2}+d_{33}E$$

To simplify the calculation, *P*≈*ε*_0_ *​*(*ε*_r​_‐1)*E* is introduced in Equation 4 followed by dividing by *E* on both sides, and Equation 5 is obtained:

(5) $$S_{33}/E=Q_{33}\varepsilon_{0}\left(\varepsilon_{r}-1\right)P+d_{33}$$

As shown in Figure 4c–f and Table S2, the EM response of the four polymers at different *E* is presented, using “*ε*_0_ *​*(*ε*_r_ *​*‐1)*P*” as the independent variable and “*S*_33_ */E*” as the dependent variable. At *E*≥*E*_c_, all polymers display a good linear relationship, and *Q*_33_, *d*_33max_ can be obtained from the linear fitting results listed in Table S3, which agree well with previously reported values [48]. This means that when the FS has completed (*E*≥*E*_c_), the IP effect and CF are the main contributions, and the sum of the three parts can finely describe the entire EM response in FEPs.At *E*<*E*_c_, the predicted result exceeds the experimental result because the FS has yet completed. Using *P_r_* obtained above *E*_c_ instead of actual *P*_r_ inevitably leads to the overestimated EM contribution from the FS effect and IP.

The actual contribution of *d*_33_ is related to the proportion of T >4, and can be estimated using *d*_33_ = *d*_33max_ × (*P*_r_’/*P*_r(max)_) (where *P*_r_’ is the remanent polarization intensity under a low electric field and *P*_r(max)_ is the saturated remanent polarization intensity of single‐crystal P(VDF‐TrFE), with a value of about 14.85 µC/cm^2^). As shown in Tables S4–S7 using *d*_33max_ instead of actual *d*_33_ to calculate *S*_33_/*E* in C‐TrFE, T‐VF and T‐FA below *E*_c_ would lead to significantly overestimated results since the difference between *d*_33max_ and actual *d*_33_ is huge. However, *d*_33max_ works well in predicting *S*_33_/*E* of T‐CFE at rather low *E*_a_ because its *d*_33max_ is really low, and the difference between *d*_33max_ and the actual *d*_33_ is negligible. Meanwhile, the much larger *Q*_33_ of T‐FA and T‐CFE than that of C‐TrFE and T‐VF suggests that the CF from T_3_GT_3_G’ to T >4 contributes more EM response than FS under a consistent electric field, while the IP effect depends on the portion of the aligned FE phase at *E*<*E*_c_. Therefore, our model provides an promising approach for establishing the correlation between the structural composition of ferroelectric polymers and their EM response at electric fields above the *E*_c_.

Apparently, the different *Q*_33_ obtained in different polymers stems from the structural composition variations; thus, quantitatively correlating *Q*_33_ to the structural parameter Δ*d/d*_0_ is essential for directly evaluating the influence of the structure compositions on the EM response. Since the *Q*_33_ *ε*_0_ *​​*(*ε*_r_ *​*‐1)*​P* part could be treated as an increment in *S*_33_/*E* for FEPs compared to the P(VDF‐TrFE) single crystal, *Q*_33_ could also be regarded as the electrostriction coefficient change induced by the varied Δ*d/d*_0_ compared to *Q*_33(s)_, which refers to the electrostriction coefficient of the P(VDF‐TrFE) single crystal. Therefore, the relationship of *Q*_33_ for the four polymers against their Δ*d/d* could be established by fitting the experimental results linearly (Figure 4g), where the slope is obtained as −54. From the literature [31], the value of *Q*_33(s)_ is assigned as −0.54 m^4^/C^2^, which is comparable to the results of our DFT calculations (Table S8 and Figures S15 and S16). Subsequently, a linear relationship between *Q*_33_ of diverse FEPs originating from structural composition‐induced modifications and *Q*_33(s)_ is established as *Q*_33_ = 100(Δ*d/d*_0_+1) × *Q*_33(s)_. Furthermore, single‐crystal P(VDF‐TrFE) exhibits a 100% T >4 conformation, with its intrinsic piezoelectric coefficient *d*_33(s)_ of approximately −30 pm/V. The piezoelectric response *d*_33_ can be calculated based on the percentage content of the T >4 conformation (denoted as *C_T > 4_*) in different FEPs. As shown in Figure 4h, *d_33_* demonstrates a strong linear correlation with *C_T > 4_*. When *C*_T > 4_ reaches 100%, the *d*_33_ value stabilizes at −30 pm/V, which is close to the intrinsic piezoelectric coefficient *d*_33(s)_ of the single crystal [31]. Eventually, the EM response of FEPs can be described as *S_33_/E* = 100(Δ*d/d*_0_+1) × *Q*_33(s)_ *ε_0​_* (*ε_r​_*‐1)*P*‐*d*_33(s)_ × *C*_T > 4_, where Δ*d*, *ε_r_*, *P*, and *C*_T > 4_ could be obtained from the XRD, BDS, *P–E* loops, and FT‐IR of different polymers. *Q*_33(s)_ = −0.54 m^4^/C^2^ and *d_33(s)_* = −30 pm/V can be considered as the standards of single‐crystal P(VDF‐TrFE). To validate the accuracy of this model, *S_33_/E* values of the other three P(VDF‐TrFE)‐based polymers, including poly(vinylidene fluoride‐co‐trifluoroethylene‐co‐chlorotrifluoroethylene) (T‐CTFE), H‐poly(vinylidene fluoride‐co‐trifluoroethylene‐co‐chlorotrifluoroethylene) (H‐T‐CTFE), and poly(vinylidene fluoride‐co‐trifluoroethylene‐co‐chlorofluoroethylene‐co‐fluoroalkyne) (T‐FA‐3%) (Supporting Information), were tested and compared with the Equation *S*_33_ */E =* 100(Δ*d/d*_0_+1) × *Q*_33(s)_ *ε_0​_* (*ε_r​_*‐1)*P*‐*d*_33(s)_ × *C*_T >4_. As shown in Tables S9–S11 and Figure 4i, the experimental results fit well with the calculated values, confirming the proposed model's accuracy.

### 2.5 Design of High‐S_33_ FEPs and Their Applications in Soft Robots

Furthermore, our proposed model *S*_33_ */E =* 100(Δ*d/d+*1) × *Q*_33(s)_ *ε_0​_* (*ε_r​_*‐1)*P* + *d*_33(s)_ × *C*_T > 4_ can give more specific guidance for the design of FEPs with large strain. Since a large *S_33_* under relatively low *E* is always required, a promising FEP should possess a large “∆*d*” along with a high “*ε_0​_* (*ε_r​_*‐1)*P*”, namely enlarging the value of 100(Δ*d/d*) × *Q*_33(s)_ *ε*_0​_(*ε*_r​_‐1)*P* term since the value of *d*_33(s)_ × *C*_T > 4_ term can be treated as a constant when *E* is over *E_c_*. Adding a third monomer with a proper steric bulk may increase the Δ*d* and disrupt the polar T >4 conformation to generate more G/G’ conformations in P(VDF‐TrFE), which may lead to the increased *ε_r_* and *P* simultaneously at low *E*, all of which favor large *S*_33_ */E*. However, if the third monomer's volume is too large, the resulting significant increase in Δ*d* value will substantially reduce the polymers’ crystallinity and permittivity. Both may hinder the formation of T_3_GT_3_G’ conformation, thus their contribution to the overall EM response. Therefore, a balanced design of the material structure is needed to maximize the “(Δ*d*/*d*_0_+1)*ε*_0_ *​*(*ε*_r​_‐1)*P*” value and achieve ultrahigh EM responses in P(VDF‐TrFE)‐based FEPs.

**FIGURE 5:**
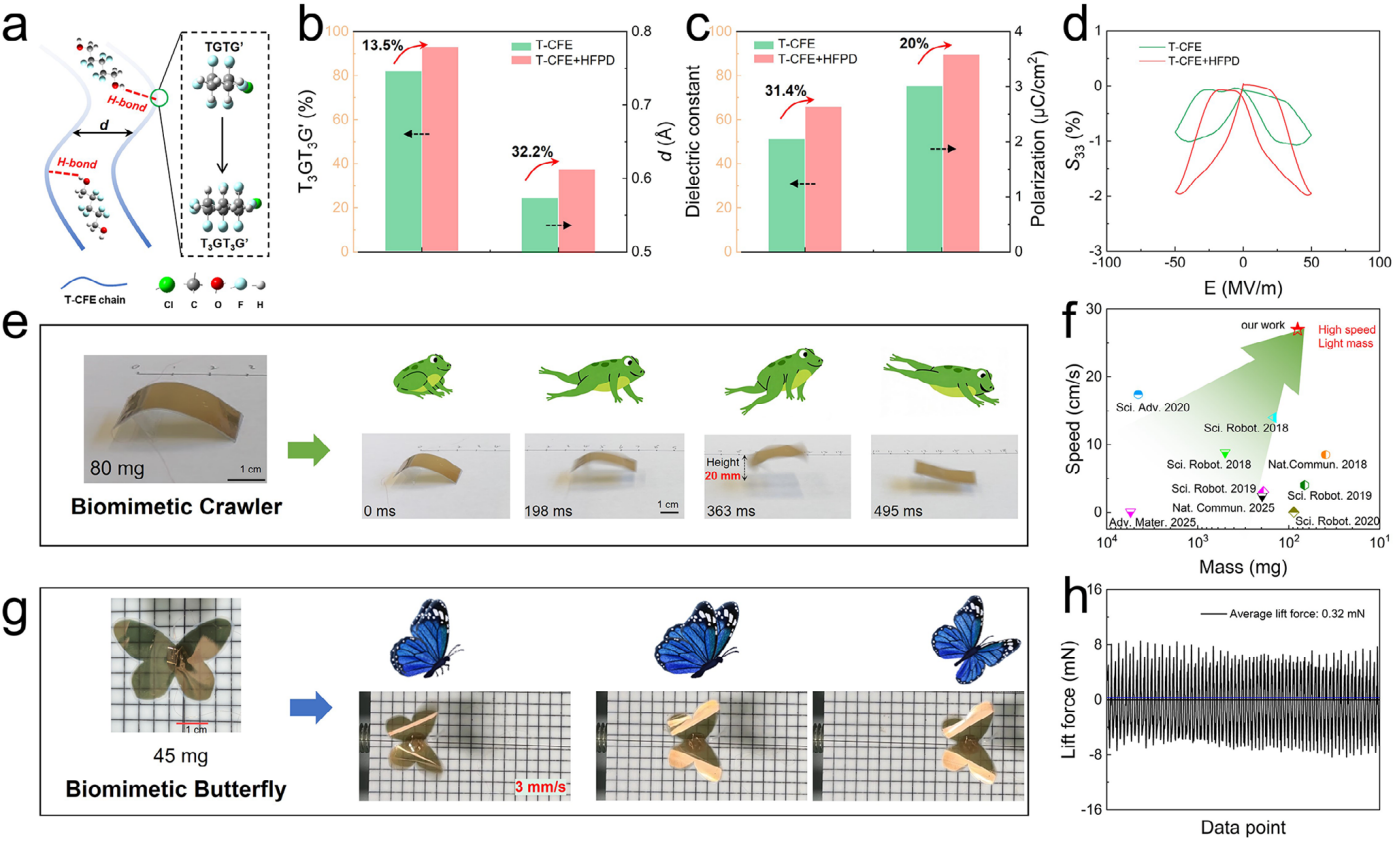
Design of high‐S_33_ FEPs and their applications in soft robots. (a) H‐bond interaction between HFPD and T‐CFE; (b) Effect of HFPD incorporation on the conformational content and *d* of T‐CFE; (c) Effect of HFPD incorporation on the dielectric constant and polarization intensity of T‐CFE; (d) Effect of HFPD incorporation on the *S*_33_ of T‐CFE; (e) Schematic diagram of the fabrication process and crawling motion of the biomimetic crawler; (f) References comparison of crawling speeds for the biomimetic crawler; (g) Schematic diagram of the fabrication process and flapping‐wing motion of the biomimetic butterfly; (h) Lift force test results of the biomimetic butterfly.

Altnatively, we chose to introduce an appropriate amount of the molecular ferroelectric hexafluoropentanediol (HFPD) into T‐CFE [49]. This enables the conformational transition from TGTG' to T_3_GT_3_G' through H‐bond interactions [50], while appropriately increasing the “*d* ” of T‐CFE. These effects synergistically enhance the value of “(Δ*d*/*d*_0_+1)*ε*_0_ *ε*_r_ *P*”, thereby achieving higher actuation performance. As shown in Figure 5a, the hydroxyl groups in HFPD can form strong hydrogen bonds with the C‐F bonds in T‐CFE(Figure S17), which facilitates the regulation of chain conformation. Molecular dynamics simulations revealed that an HFPD content of 3% yields the optimal proportion of the T_3_GT_3_G' conformation (Figure S18). As illustrated in Figure 5b,c, the incorporation of HFPD increases the T_3_GT_3_G' content in T‐CFE to 92%, the “*d* ” to 0.62, the dielectric constant to 67, and the polarization intensity to 3.6 (Figures S19–S22). Overall, the “(Δ*d*/*d*_0_)*ε*_0_ *ε*_r_ *P*” value increased by approximately 108%. As shown in Figure 5d, the *S*_33_ of T‐CFE is about 1.0% at 50 MV/m, while the introduction of HFPD achieves a *S*_33_ of about 2.0%, representing an increment of 100%. This result aligns closely with the preceding calculations, confirming the feasibility of the material design.

This tailorable, large actuation strain provides a convenient pathway for fabricating high‐performance soft robots. As demonstrated in Figure 5e,f, the composite film was used to fabricate a biomimetic crawler. Due to the higher actuation strain, the crawler achieved a locomotion speed of 27 cm/s under actuation conditions of 20 MV/m and 16 Hz (Video S1), outperforming most existing soft robots while also having a lower self‐weight (Figure 5f and Table S12). Furthermore, the application of the composite film in a biomimetic butterfly was demonstrated. As shown in Figure 5g, the motion of the butterfly strongly depends on large deformations of the flapping wings. Benefiting from the high actuation strain, a motion pattern resembling butterfly wing flapping was achieved under actuation at 40 MV/m and 4 Hz (Video S2). The lift force generated by the biomimetic flapping wing under these conditions was further measured to be approximately 0.32 mN, resulting in a thrust‐to‐weight ratio of 0.71. Although this ratio still falls below the requirements for take‐off and the performance of current typical micro flapping‐wing vehicles [51], this direct‐drive mode greatly simplifies the vehicle's structure, and outperform soft robots utilizing the same actuation mode [52]. Future optimization of the material's actuation performance could potentially achieve a thrust‐to‐weight ratio greater than 1, enabling take‐off.

## 3 Conclusion

To better characterize the EM response of structurally complex FEPs, we introduced a key structural parameter—the relative interplanar spacing change (Δ*d*/*d*_0_) of FEPs compared to single‐crystal P(VDF‐TrFE) *d*_0_—to quantitatively correlate multiple physical responses with EM performance across different FEPs. We have found that there is a quantitative relation *Q*_33_ = 100 (Δ*d*/*d*_0_+1) × *Q*_33(s)_ between the *Q*_33_ of different FEPs and their structural compositions, revealing the dependence of FEPs' EM response on their structural composition. Furthermore, guided by model, the regulation of the T‐CFE structure and electrical parameters was achieved by adding an appropriate amount of HFPD, resulting in a thickness strain of 2.0% at 50 MV/m. This was applied to the fabrication of high‐performance soft robots, including a biomimetic crawler with a crawling speed of up to 27 cm/s and a biomimetic butterfly with a lift‐to‐weight ratio of 0.71. Our result offers a new theoretical guidance for designing next‐generation flexible FEPs with a higher EM response.

## 4 Methods

### 4.1 Synthesis of T‐VF, T‐FA

The pristine P(VDF‐TrFE‐CFE) (T‐CFE) terpolymer with the composition of VDF/TrFE/CFE = 65/35/7 mol% was purchased from Arkema. The VF and FA were introduced by fully hydrogenation and elimination reaction from T‐CFE as schematically shown in Figure S1. For the fully hydrogenation reaction, using tris(trimethylsilyl)silane (TTMSS) as a reducing agent (purchased from Aladdin), 2,2'‐azobis(2‐methylpropionitrile) (AIBN) as a free radical initiator, and anhydrous tetrahydrofuran (THF) as the solvent. A typical reaction as follows, T‐CFE (1 g), AIBN (40 mg), and TTMSS (1 mL) were added to a 100 mL reaction flask (under anhydrous, oxygen‐free and nitrogen atmosphere) and reacted at 65°C for 24 h. Finally, the product was precipitated in deionized water, yielding a white terpolymer, named T‐VF. The H‐CTFE is synthesized by same method, more information in our previous result [33]. For the elimination reaction, using triethylamine (TEA) as a base and N‐Methyl‐2‐pyrrolidone (NMP) as the solvent, T‐CFE (1 g), NMP (40 mL), and TEA (0.5 mL) were added to a 100 mL reaction flask and reacted at 60°C for 24 h. The product was then precipitated in a mixed solution of deionized water and hydrochloric acid (v:v = 99:1), yielding a brown ternary polymer, named T‐FA. (Reducing the reaction time to 12h can obtain the T‐FA‐3%). The chemical composition of all polymers was confirmed by proton nuclear magnetic resonance (^1^H NMR) and fluorine nuclear magnetic resonance (^19^F NMR) using dimethyl sulfoxide‐*d*_6_ as the solvent. Unless otherwise specified, all other reagents were purchased from MERDA with AR grade, and they were used directly without further purification.

### 4.2 The Preparation of Films and Soft Robots

First, all polymers were dissolved in dimethylformamide (DMF) at room temperature with a concentration of 0.1 g/3 mL to form homogeneous solution. For T‐CFE+HFPD, the HFPD was added at 3 wt.%. Then, the homogeneous solution was dropped onto a 10 cm × 10 cm clean glass substrate in an oven for 15 h at 60 °C. After the DMF has completely evaporated, the polymers were transferred to a vacuum oven and annealed at 120 °C for 24 h. Last, the polymer films were immersed in deionized water to be peeled from the substrate for the next characterization. Besides, for the TF‐IR test, the solution concentration should be 0.1 g/5 mL, all the other conditions are the same. For the fabrication of soft robots, the polymer film is cut to the required dimensions.

### 4.3 Characterization

#### 4.3.1 Structure Characterization

X‐ray Diffraction (XRD), Fourier Transform Infrared Spectrometer (FT‐IR) and Proton/ Fluorine Nuclear Magnetic Resonance (^1^H NMR and ^19^F NMR)

The XRD data were collected by Bruker (D8 ADVANCE) for the 2*θ* from 10° to 50°. The FT‐IR data were measured by Bruker (INVENIO) with ATR model. The conformation content was calculated by $F_{i}=\frac{A_{i}}{A_{TGTG^{\prime }}+A_{T>4\;}+A_{T3GT3G^{\prime }}}\;,$where *A_i_* can be *A_TGTG’_*, *A_T>4_* and *A_T3GT3G'_*, the absorbances of TGTG′, T>4 and T_3_GT_3_G' is 614, 1288, 772 cm^−1^, respectively (assuming the absorption coefficients for the respective wavenumbers are the same). The ^1^H and ^19^F NMR data were collected by 400MHz JOEL (JNM‐ECZ400S/L1) after 32 scans. All data were normalized with respect to the film thickness.

#### 4.3.2 Performance Characterization of Soft Robots

The motion processes of the biomimetic crawler and the biomimetic butterfly were recorded using a mobile phone (Huawei Mate 70). The driving voltage for the flapping wing is generated by a high‐voltage power supply (RK2674B), and the thrust and torque are measured by a six‐component balance. The six‐component balance used in this test platform is the NANO17 Ti from ATI (USA). This sensor has a maximum sampling frequency of 3000Hz, with a force measurement resolution of 1/341N and a torque measurement resolution of 5/364N·m, which can meet the requirements for measuring the flapping‐wing lift.

### 4.4 Electrical Performance

Before the test, the gold was sputtered on the polymer films as the electrodes for all electrical and strain measurements.

#### 4.4.1 Dielectric Properties (Electrodes Area was 0.785 cm^2^)

The dielectric properties were measured by NOVOCONTROL (Concept 80) under 1V from 10^2^ Hz to 10^6^ Hz at room temperature and the temperature dependent dielectric data were collected from −20°C to 120°C with a temperature rise rate 4 K/min.

#### 4.4.2 Polarization‐Electric Loops (P‐E Loops, Electrodes Area was 0.069 cm^2^)

The *P*–*E* loops data were measured by Premiere II ferroelectric tester (Radiant Technologies) at 1Hz from 50 MV/m to 200 MV/m (a triangular waveform AC electric fields).

#### 4.4.3 Infrared Spectrum Under Electric Fields (Electrodes Area was 0.785 cm^2^)

The data were measured by Bruker (INVENIO) with TR model, and the voltage was applied to film via a voltage amplifier (RK2674B) under a DC voltage ramp of 20MV/m connected to the copper wires.

#### 4.4.4 The Strain Measurement (S_33_, Electrodes Area was 0.196 cm^2^)

The strains were measured via a non‐contact fiber optic measuring and the sensor is MIT‐2100 with a 2032R probe. A step ramp of 20 MV/m and a testing frequency of 1 Hz are used under a triangle wave electric field.

## Author Contributions

**Ba Qin**: conceptualization, validation, writing – original draft, investigation, software. **Guotong Ding**: methodology. **Wanli Xing**: methodology. **Xiaoyu Yang**: data curation. **Wenxuan Li**: formal analysis. **Zhemin Chen**: formal analysis. **Shaobo Tan**: writing – review and editing, writing – original draft, resources. **Xiaoyong Wei**: conceptualization, visualization. **Zhicheng Zhang**: conceptualization, funding acquisition, writing – original draft, writing – review and editing, project administration, supervision.

## Funding

The National Key Research & Development (R&D) Program of China (Grant No. 2023YFB3208400). Major Research Plan of the National Natural Science Foundation of China (Grants 92066204). National Natural Science Foundation of China (Grants 52073225).

## Conflicts of Interest

The authors declare no conflicts of interest.

## Supporting information




**Supporting File 1**: advs75884‐Sup‐0001‐SuppMat.docx.

**Supporting File 2**: advs75884‐Sup‐0002‐VideoS1.mp4.

**Supporting File 3**: advs75884‐Sup‐0003‐VideoS2.mp4.

## Data Availability

The data that support the findings of this study are available from the corresponding author upon reasonable request.
