# Supplementary material for: Quantifying Structure–Property Relationships in Ferroelectric Polymers Toward High‐Performance Soft Robots
Source: Adv Sci (Weinh). 2026 May 29;13(47):e75884. doi: 10.1002/advs.75884 (PMC13335897; doi:10.1002/advs.75884)
Supplement: Supplementary file 1 [file ADVS-13-e75884-s003.docx]

Supporting Information

**Quantifying Structure–Property Relationships in Ferroelectric Polymers toward High-Performance Soft Robots**

*Ba Qin^1^, Guo-Tong Ding^1^, Wan-Li Xing^1^, Xiao-Yu Yang^1^, Wen-Xuan Li^1^, Zhe-Min Chen^1^, Shao-Bo Tan^1^*, Xiao-Yong Wei^2^*, Zhi-Cheng Zhang^1^**

**Supporting text**

**Density Functional Theory Calculations**

**The Δenergy of different conformations**

In order to compare the ease of transition from the TGTG' and T_3_GT_3_G’ conformations to the TTTT conformation, we constructed a polymer chain containing 10 VDF monomers with VDF as the basic repeating unit, and simulated different initial conformations by adjusting the dihedral angles between adjacent C-C bonds. For the T conformation, the dihedral angle is 180°, and for the G and G' conformations, the dihedral angles are 60° and -60°, respectively. Therefore, for the TGTG' conformation, the dihedral angles are 180°, 60°, 180°, and -60°, while for the T3GT3G' conformation, the dihedral angles are 180°, 180°, 180°, 60°, and 180°, 180°, 180°, -60°. First, the above PVDF chain was adjusted to the fully TGTG’ conformation, and then the content of T conformations was gradually adjusted, transitioning from the TGTG’ conformation to the T_3_GT_3_G’ conformation with contents of 25%, 50%, 75%, and 100%, respectively. Finally, a TTTT chain was constructed to represent the *all-trans* structure. Therefore, a total of six chains were constructed, each containing different conformations, as follows:

TGTG’(100%),TGTG’(75%)+T_3_GT_3_G’(25%),TGTG’(50%)+T_3_GT_3_G’(50%),TGTG’(25%)+ T_3_GT_3_G’(75%), T_3_GT_3_G’(100%), TTTT(100%). Then, used DFT-B3LYP and the 3-21G basis set, as implemented in the Gaussian 09 software, to calculate the energy of different chains.

**Molecular Dynamics (MD) Simulations**

Molecular dynamics (MD) simulations were conducted to study the structural and dynamic properties of the polymer system. Polymer force field parameters were generated using the Sobtop program with the General Amber Force Field (GAFF) for bonded and non-bonded interactions. Atomic partial charges were assigned via the MMFF94 scheme, known for its balanced accuracy and efficiency in organic/polymeric systems. Initial configurations were built with Packmol to ensure reasonable molecular distribution. All simulations were performed in GROMACS. To calculate the influence of HFPD content on the conformation of TP chains, molecular chains containing 20 repeating units (VDF:TrFE:CFE = 6:3:1) were constructed using Materials Studio, totaling 20 chains. HFPD was then added at mass fractions of 1 wt%, 3 wt%, and 6 wt%, respectively. After energy minimization, the content of the T_3_GT_3_G’ conformation in randomly selected chains from each system was statistically analyzed^[1]^.

**IRI+VMD program**

The Interaction Region Indicator (IRI) is a straightforward tool that can graphically illustrate various interactions within a chemical system^[2]^. The green areas between the chains represent the interaction strength between adjacent molecular chains, and the darker of the color indicates the stronger of the interaction. The RDG function is combined to facilitate comparison, where the more points near 0.005 indicate the stronger interactions. It can be intuitively observed that, the introduction of FA and CFE leads to weakened interactions than C-TrFE, while the introduction of VF strengthens the interactions, making the FE switching require higher energy to take place, which is consistent with the observations made in Figure 2b. Therefore, the ∆*d* caused by the steric volume of the monomers is dominating the electrical response of polymers by tailoring the both the interaction in polymer chains and that inter polymer chains.

**Calculation of Q_33_**

All geometries given below were optimized at ωB97XD/6-311+G(2d) level using Gaussian 16 C.01 with default setting. No imaginary frequency was found. The external electric field was applied in Y direction. For example, the case of EEF=0.001 a.u. corresponds to using the keyword of field=Y+10 in Gaussian^[3]^.

**Supplementary Figures**

Figure S1. The reaction scheme of full Hydrogenation and Elimination in T-CFE to obtain T-VF and T-FA terpolymers.

Figure S2. The ^19^F NMR spectra of C-TrFE and terpolymers.

Figure S3. The FT-IR spectra of four polymers.

Figure S4. The XRD spectra of four polymers.

Figure S5. The FT-IR of C-TrFE under different electric fields.

Figure S6. The FT-IR of T-VF(a) and T-FA(b) under different electric fields.

Figure S7. The P-E loops and current for four polymers at 200MV/m.

Figure S8. The P-E loops of C-TrFE and Terpolymers at various electric fields.

Figure S9. Trend of polarization values with increasing electric fields.

Figure S10. The temperature-dependence dielectric constant of for polymers.

Figure S11. The fitting lines of dielectric relaxation strength (DRS) by equation *lnf = lnf*_0_*-(T*_0_*/T*_p_*)^p^*.

Figure S12. The *S*_33_-*E* curves of four polymers at various electric fields. From (a) to (d), the curves are C-TrFE, T-VF, T-FA and T-CFE.

Figure S13. The S_33_ of four polymers at various electric fields.

Figure S14. The relationship curves of *S*_33_ with *P*^2^ for four polymers at 200MV/m.

Figure S15. The Deformation of P(VDF-TrFE) molecular chains under electric field

Figure S16. Schematic diagram of strain of P(VDF-TrFE) unit under electric field

Figure S17. Temperature-dependent infrared spectroscopy confirmed the presence of hydrogen bonds

Figure S18. Schematic diagram of molecular dynamics simulation of the interaction between HFPD and T-CFE and the conformational evolution

Figure S19. The addition of HFPD to T‑CFE induces an increase in the content of the T_3_GT_3_G’ conformation.

Figure S20. The addition of HFPD to T‑CFE leads to a slight increase in “*d*”

Figure S21. The addition of HFPD to T‑CFE leads to increased dielectric constant

Figure S22. The addition of HFPD to T‑CFE leads to increased polarization intensity

**Supplementary Tables**

Table S1. The ^19^F NMR belongingness in C-TrFE, T-VF, T-FA and T-CFE.

| 5C sequences | Designation | Chemical Shift  (ppm) |
| --- | --- | --- |
| CF_2_CH_2_CF_2_CH_2_CF_2_ | VDF-VDF | -93.2 |
| CF_2_CH_2_CF_2_CHFCF_2_ | VDF-TrFE | -107.8 |
| CF_2_CH_2_CFHCH_2_CF_2_ | VDF-VF | -180.1 |
| CF_2_CH=CFCH2CF_2_ | VDF-FA | -87.6 |
| CF_2_CH_2_CFClCH_2_CF_2_ | VDF-CFE | -98.7 |

Table S2. The *S*_33_*/E* result of different FEPs and their corresponding Δ*d*, *ε_r_* and *P*.

|  | ^a^*S_33_/E* (pm/V) | Δ*d* (Å) | *ε_r_* (1k Hz) | *P* (μC/cm^2^*)* |
| --- | --- | --- | --- | --- |
| C-TrFE | -65 | 0.24 | 12 | 11.0 |
| T-VF | -40 | 0.19 | 10 | 6.5 |
| T-FA | -129 | 0.40 | 28 | 8.7 |
| T-CFE | -310 | 0.56 | 50 | 9.4 |

Note: a, the “*S*_33_/*E*” is calculated at 200MV/m.

Table S3. The *Q_33_* and *d*_33(max)_ of four polymers.

|  | *Q*_33_ (m^4^/C^2^) | *d*_33(max)_ (pm/V) |
| --- | --- | --- |
| C-TrFE | -3.5 | -28.0 |
| T-VF | -2.9 | -24.8 |
| T-FA | -5.5 | -15.6 |
| T-CFE | -7.5 | -1.2 |

Table S4. The “*S*_33_/*E*” result of C-TrFE at different methods.

|  | Test value  (pm/V) | Calculated value (pm/V) | ^a^Modified value (pm/V) |
| --- | --- | --- | --- |
| 40MV/m | -10.1 | -30.4 | -2.9 |
| 50MV/m | -21.2 | -36.4 | -9.5 |
| 60MV/m | -36.1 | -45.9 | -22.8 |
| 80MV/m | -52.8 | -54.3 | -36.5 |

Note: a. The modified value is calculated by: *S*_33_/*E* = “Calculated value” + *d*_33max_× (*P*_r_’/*P*_r(max)_)-*d*_33max_. Where *d*_33max_ is -28.0 pm/V.

Table S5. The “*S*_33_/*E*” result of T-VF at different methods.

|  | Test value  (pm/V) | Calculated value (pm/V) | ^a^Modified value (pm/V) |
| --- | --- | --- | --- |
| 40MV/m | -5.1 | -26.3 | -1.6 |
| 50MV/m | -6.3 | -26.5 | -2.2 |
| 60MV/m | -8.1 | -26.9 | -2.7 |
| 80MV/m | -10.7 | -27.8 | -4.3 |

Note: a. The modified value is calculated by: *S*_33_/*E* = “Calculated value” + d_33max_× (*P*_r_’/*P*_r(max)_)-*d*_33max_. Where *d*_33max_ is -24.8 pm/V.

Table S6. The “*S*_33_/*E*” result of T-FA at different methods.

|  | Test value  (pm/V) | Calculated value (pm/V) | ^a^Modified value (pm/V) |
| --- | --- | --- | --- |
| 40MV/m | -40.1 | -54.0 | -39.4 |
| 50MV/m | -52.1 | -60.7 | -46.5 |
| 60MV/m | -63.3 | -68.1 | -54.1 |
| 80MV/m | -75.3 | -77.5 | -63.9 |

Note: a. The modified value is calculated by: *S*_33_/*E* = “Calculated value” + *d*_33max_× (*P*_r_’/*P*_r(max)_)-*d*_33max_. Where *d*_33max_ is -15.6 pm/V.

Table S7. The “*S*_33_/*E*” result of T-CFE at different methods.

|  | Test value  (pm/V) | Calculated value (pm/V) | ^a^Modified value (pm/V) |
| --- | --- | --- | --- |
| 40MV/m | -93.3 | -93.3 | -92.3 |
| 50MV/m | -115.8 | -115.8 | -115.4 |
| 60MV/m | -140.2 | -140.2 | -139.3 |
| 80MV/m | -177.4 | -177.5 | -177.1 |

Note: a. The modified value is calculated by: *S*_33_/*E* = “Calculated value” + *d*_33max_× (*P*_r_’/*P*_r(max)_)-*d*_33max_. Where *d*_33max_ is -1.2 pm/V.

Table S8. Comparison of Structural and Electrical Parameters of P(VDF-TrFE) unit without and applied electric fields

|  | Without E | Applied E |
| --- | --- | --- |
| unit numbers | 7 | 7 |
| chain volume (Å^3^)  number density | 412.37032  1.60050×10^28^ | 412.4101  1.69734×10^28^ |
| height (Å)  α angel (°) | 0.863781356  110.99892 | 0.860165094  111.32832 |
| dipole moment (C×m) | 5.56486×10^−30^ | 5.43032×10^−30^ |
| P (C/m^2^) | 0.089065785 | 0.092170891 |

Applied E, the *S*_33_:

$S_{33}=(h_{E}-h_{0})/h_{0}$= −0.42%

Applied *E*, the *P_E_*:

$P_{E}=n*<\mu>$= 0.092 C/m^2^

Thus:

$Q_{33}=S_{33}/P_{E}^{2}$= −0.50 m^4^/C^2^

This calculated result is very close to our fitting result of *Q*_33(s)_ = −0.54 m^4^/C^2^ (the reason for the slightly smaller calculated value may be that the statistically obtained number density is larger, leading to a lower calculation of *P*). Therefore, combined with DFT calculations, we have verified the rationality of *Q*_33(s)_ = −0.54 m^4^/C^2^ for P(VDF-TrFE) single crystals.

Table S9. The calculated and test result of *S*_33_/*E* in T-CTFE polymer.

|  | Measurable structural and electrical parameters | | | | *S*_33_*/E* (pm/V) | |
| --- | --- | --- | --- | --- | --- | --- |
| fields | Δ*d*(Å) | *ε_r_* | *P*(μC/cm^2^) | *C*_T>4_ | calculated | test |
| 200MV/m | 0.58 | 32 | 8.3 | 0.02 | 177 | 180 |
| 150MV/m | 0.58 | 32 | 6.4 | 0.02 | 134 | 140 |
| 100MV/m | 0.58 | 32 | 5.9 | 0.02 | 124 | 127 |

Table S10. The calculated and test result of *S*_33_/*E* in T-FA-3% polymer.

|  | Measurable structural and electrical parameters | | | | *S*_33_*/E* (pm/V) | |
| --- | --- | --- | --- | --- | --- | --- |
|  | Δ*d* | *ε_r_* | *P* | *C*_T>4_ | calculated | test |
| 200MV/m | 0.53 | 43 | 9.4 | 0.05 | 250 | 253 |
| 150MV/m | 0.53 | 43 | 6.8 | 0.05 | 181 | 183 |
| 100MV/m | 0.53 | 43 | 6.0 | 0.05 | 160 | 163 |

Table S11. The calculated and test result of *S*_33_/*E* in H-T-CTFE polymer.

|  | Measurable structural and electrical parameters | | | | *S*_33_*/E* (pm/V) | |
| --- | --- | --- | --- | --- | --- | --- |
|  | Δ*d* | *ε_r_* | *P* | *C*_T>4_ | calculated | test |
| 200MV/m | 0.60 | 13 | 4.9 | 0.03 | 43 | 45 |
| 150MV/m | 0.60 | 13 | 4.2 | 0.03 | 36 | 40 |
| 100MV/m | 0.60 | 13 | 3.6 | 0.03 | 29 | 34 |

Table S12. Comparison of Mass and Speed of soft crawlers

| Mass (mg) | Speed (cm/s) | Ref |
| --- | --- | --- |
| 4500 | 17.4 | ^[4]^ |
| 190 | 3 | ^[5]^ |
| 88 | 0.04 | ^[6]^ |
| 148 | 14 | ^[7]^ |
| 67 | 4 | ^[8]^ |
| 39.4 | 8.5 | ^[9]^ |
| 500 | 8.846 | ^[10]^ |
| 5470 | 0.075 | ^[11]^ |
| 196 | 2.4 | ^[12]^ |
| 80 | 27 | Our work |

From Table S10 to S12, according to our model, it is obvious that there is a good agreement between the calculated results of different FEPs and the actual measurements. The accuracy of the model is proved.

**Supporting videos**

**S video 1**

Biomimetic Crawler

**S video 2**

Biomimetic Butterfly

References:

[1]	X. Li, S. He, Y. Jiang, J. Wang, Y. Yu, X. Liu, F. Zhu, Y. Xie, Y. Li, C. Ma, Z. Shen, B. Li, Y. Shen, X. Zhang, S. Zhang, C.-W. Nan, *Nat. Commun.* **2023**, *14*, 5707.

[2]	T. Lu, Q. Chen, *Chemistry–Methods* **2021**, *1*, 231.

[3]	T. Lu, Q. Chen, *Chem. Phys. Chem* **2021**, *22*, 386.

[4]	Y. Tang, Y. Chi, J. Sun, T.-H. Huang, O. H. Maghsoudi, A. Spence, J. Zhao, H. Su, J. Yin, *Sci. Adv.* **2020**, *6*, eaaz6912.

[5]	X. Ji, X. Liu, V. Cacucciolo, M. Imboden, Y. Civet, A. El Haitami, S. Cantin, Y. Perriard, H. Shea, *Sci. Robot.* **2019**, *4*, eaaz6451.

[6]	X. Yang, L. Chang, N. O. Pérez-Arancibia, *Sci. Robot.* **2020**, *5*, eaba0015.

[7]	S. D. de Rivaz, B. Goldberg, N. Doshi, K. Jayaram, J. Zhou, R. J. Wood, *Sci. Robot.* **2018**, *3*, eaau3038.

[8]	Y. Wu, J. K. Yim, J. Liang, Z. Shao, M. Qi, J. Zhong, Z. Luo, X. Yan, M. Zhang, X. Wang, R. S. Fearing, R. J. Full, L. Lin, *Sci. Robot.* **2019**, *4*, eaax1594.

[9]	H. Lu, M. Zhang, Y. Yang, Q. Huang, T. Fukuda, Z. Wang, Y. Shen, *Nat. Commun.* **2018**, *9*, 3944.

[10]	G. Gu, J. Zou, R. Zhao, X. Zhao, X. Zhu, *Sci. Robot.* **2018**, *3*, eaat2874.

[11]	Q. Zheng, L. Xin, Q. Zhang, F. Shen, X. Lu, C. Cao, C. Xin, Y. Zhao, H. Liu, Y. Peng, J. Luo, H. Guo, Z. Li, *Adv. Mater.* **2025**, *37*, 2417380.

[12]	F. Zhao, J. Zhang, H. Tian, R. Zhu, L. Sun, W. Dou, H. Chen, Z.-G. Ye, C. Yi, X. Chen, *Nat. Commun.* **2025**, *16*, 10301.
